# Personal

**Published:** 1916-04

**Authors:** 


### PERSONAL.

---

**Announcement of removal, travel, and other matters of interest are requested. Please report errors in the listing of any physician in the State and other directories, that we may co-operate with the State Society in securing a correct list.**

---

Dr. Frederick J. Haller of Buffalo returned from Florida the first of March.

---

Dr. A. L. Benedict of Buffalo would be greatly obliged for information as to Indian village sites, earth-works, graves and finds of relics, in western N. Y. and eastern Ontario.

---

Dr. Monroe Manges of Buffalo returned from a trip to Florida and the West Indies, about the first of April.

---

Dr. Lawrence G. Hanley of Buffalo, visited in Connecticut about the first of March.

---

Dr. Milton E. Bork, Buffalo 1915, has been substituting on the staff of Bellevue Hospital and has received second appointment on the House Staff of the Ear, Nose and Throat Dept. of the N. Y. Post Graduate Hospital.

Dr. Eloise Walker of Binghamton spent a week in Buffalo last month.

Dr. Richard J. Gould of Buffalo announces his removal to 1355 Main Street, near Utica.

Dr. Alfred W. Armstrong of Canandaigua will limit his practice to Surgery and Diagnosis after April 1.

Dr. John L. Eckel has removed his offices and residence to 476 Franklin Street, near Allen.

Dr. W. C. Heussy (Buffalo 1895) has moved from 708 Leary Bldg. to 902 Boren Avenue, Seattle.

The Martin H. Smith Co. announce their removal from 105 Chambers Street to 150-156 Lafayette Street, New York City.

Dr. A. W. Palmer of Buffalo returned from a southern trip March 18.

